# Folic Acid Supplementation during Pregnancy Induces Sex-Specific Changes in Methylation and Expression of Placental 11β-Hydroxysteroid Dehydrogenase 2 in Rats

**DOI:** 10.1371/journal.pone.0121098

**Published:** 2015-03-20

**Authors:** Reyna Penailillo, Angelica Guajardo, Miguel Llanos, Sandra Hirsch, Ana Maria Ronco

**Affiliations:** Laboratory of Nutrition and Metabolic Regulation, Human Nutrition Unit, Institute of Nutrition and Food Technology (INTA), Universidad de Chile, Santiago, Chile

## Abstract

In the placenta, 11β-hydroxysteroid dehydrogenase type 2 (11β-HSD2) limits fetal glucocorticoid exposure and its inhibition has been associated to low birth weight. Its expression, encoded by the *HSD11B2* gene is regulated by DNA methylation. We hypothesized that maternal diets supplemented with folic acid (FA) during pregnancy modify the expression of placental *HSD11B2* through gene methylation. Wistar rats were fed with high (8 mg/kg) or normal low (1mg/kg, control) levels of FA during pregnancy. Concentrations of mRNA and protein in placentas were determined by qRT-PCR and Western blot respectively. Methylation in five CpG sites of the placental *HSD11B2* promoter (−378 to −275) was analyzed by bacterial cloning and subsequent sequencing. In the FA-supplemented group, mRNA and protein levels of 11β-HSD2 decreased by 58% and increased by 89%, respectively, only in placentas attached to males. In controls, most CpG sites were not methylated except for the CpG2 site which was 80% methylated. CpG2 methylation level increased under the FA treatment; however, only in placentas attached to females was this increase significant (113%). This change was not related to *HSD11B2* expression. Fetal weight of females from FA- supplemented mothers was 6% higher than females from control mothers. In conclusion, this is the first study reporting that FA over supplementation during pregnancy modifies the placental *HSD11B2* gene expression and methylation in a sex-dependent manner, suggesting that maternal diets with high content of FA can induce early sex-specific responses, which may lead to long-term consequences for the offspring.

## Introduction

During pregnancy, the fetus requires favorable nutritional and hormonal environment for optimal growth. Folic acid (FA), a water-soluble vitamin B, is an essential nutrient during gestation for DNA methylation, synthesis and repair, and it is well known to reduce the risk of neural tube defect (NTD) in the new-born [1,2]. Its consumption has significantly increased in several countries [3], and could exceed the tolerable levels (UL: 1000 μg FA/day) [4] raising questions about the potential deleterious consequences of such elevated intake on methylation of specific genes, affecting their expression and predisposing to long term consequences [5]. Women at reproductive age who are planning to get pregnant are recommended to take FA supplementation [6,7] to reduce the risk of NTD during fetal development [8,9].

The placenta is the main interface between the fetus and the mother and regulates intrauterine development through the supply of nutrients, hormones and oxygen [10]. The placenta also plays a major role in the protection of the fetus to glucocorticoids (GC) overexposure [11,12]. Although these steroids have a fundamental role in fetal development and maturity, an excess of GC may have deleterious effects in the fetus and may explain, at least in part, how fetal programming arise [13,14]. Accordingly, excessive GC exposure is associated with intrauterine growth restriction (IUGR) in both humans and animals, and has been reported to be involved in fetal programming of diseases expressed during adulthood [12,14].

Glucocorticoids influence the developing fetus by binding to GC receptor (GR), which subsequently acts as a transcription factor to affect the expression of target genes. The access of GC to GR is modulated by the 11β-hydroxysteroid dehydrogenase enzymes (11β-HSD1 and 11β-HSD2, encoded by the *HSD11B2* gene). The placenta expresses high levels of 11β-hydroxysteroid dehydrogenase type 2 (11β-HSD2), specifically in the syncytiotrophoblast [15,16], and converts active cortisol (corticosterone in rodents) to inactive cortisone (11-dehydrocorticosterone in rodents), protecting thus the fetus from an excess of GC [11]. It has been demonstrated that placental genes and particularly 11β-HSD2 are expressed in a sex dependent manner [17]. Such sex differences are mainly related to placental response to GC exposure consistent with the sex-biasing influences on gene networks [18,19].

The expression of *HSD11B2* is regulated by epigenetic mechanisms like DNA methylation of its promoter through DNA methyltransferases (DNMTs) [15,20]. Any disruption in the placental epigenome may potentially affect the developing fetus leading to long-term functional alterations throughout life [21–25]. Based on this evidence and previous studies suggesting that placental DNA methylation could modify fetal epigenetic marks [1], the effect of nutrients containing methyl groups or those affecting the metabolism of 1-C compounds during pregnancy on the expression and methylation of glucocorticoid-related genes like *HSD11B2* in placental tissue remains to be elucidated.

Placental 11β-HSD2 is the most important enzyme regulating fetal exposure to GC. Since dietary factors may alter the expression of placental *HSD11B2* through epigenetic mechanisms, our working hypothesis is that an oversupply of FA in the diet of pregnant rats alters placental methylation and expression of *HSD11B2*. We therefore analyzed mRNA, protein and methylation levels of *HSD11B2* in placentas of rats supplemented with high or normal low levels of FA during pregnancy.

## Material and Methods

### Animals and diets

All procedures were performed according to the guidelines of the American Veterinary Medical Association (AVMA) [26], and approved by our local Bioethics Committee for Animal Experimentation at the Institute of Nutrition and Food Technology (INTA), University of Chile, Santiago, Chile. Virgin female Wistar rats (70 days, 200–250 g weight) were caged with mature male breeders (2 x 1). Mating was confirmed by the presence of a vaginal copulation plug (gestation day 0). At this time, pregnant rats (n = 12) were divided into 2 groups according to FA contents in the diet (AIN-93G diet; Research Diets, Inc., NJ, USA), which was maintained during the whole pregnancy period. Group 1-FA (control, diet D10040902M) and 8-FA (diet D10040903M) received a diet containing 1 or 8 mg of FA per kg of food respectively. The first diet is considered normal low FA intake for rodents [27] and 8 mg/kg FA represents a high FA diet [28]. Paternal diets contained 1mg/kg FA. During the experimental period the animals were housed one per cage and were maintained at 22°C with 12:12 hour light-dark. Food and water were available ad libitum and replenished daily by the animal care staff. At day 20 of gestation, the mothers were weight and fetuses were extracted by caesarean. All placentas were collected, weighted and frozen immediately at −80°C for subsequent analyses. Six pregnant rats were selected per group; fetal characteristics were determined in male and female offspring (n = 38 and 32 respectively). For the molecular studies (mRNA and WB) and FA measurements, we analyzed one whole placenta per sex and per litter from each of the six pregnant rats.

### DNA extraction, Na-bisulfite treatment and Methylation studies

**Fig 1:**
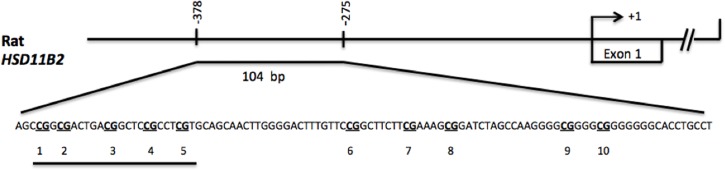
Schematic representation of rat *HSD11B2* promoter. The diagram indicates nucleotide −378 to nucleotide −275 with respect to the transcription initiation site (arrow at +1) next to the exon 1 of the gene. The described sequence shows the amplicon of 104 bp containing 10 CpG sites. Line below the sequence indicates the first 5 CpG sites that were analyzed for the methylation studies.

Genomic DNA was extracted from placental samples using the QIAmp micro Kit (Qiagen, USA) following the manufacturer’s instructions, and treated with sodium bisulfite to convert non-methylated cytosine to uracil using EZ DNA Methylation Gold Kit (Zymo Research Corp., USA). The bisulfite reaction was performed on 0.5 μg DNA. PCR was done using specific primers (Assay ADS462; Rat HSD11B2 promoter ENSRNOT00000023130: −378 to −275 from start codon designed by EpigenDX, USA). The PCR products from bisulfite-treated genomic DNA were cloned into a pGEM-T Easy Vector (Promega, USA) following manufacturer’s instructions. Six to eight colonies from PCR cloning were selected and grown overnight in LB-Ampicillin medium at 37°C. The plasmid DNA was prepared using the Wizard plus SV Minipreps DNA purification system (Promega, USA), and sent to Macrogen (Korea) for sequentiation [29]. The PCR amplicon of 104 bp contained 10 CpG sites; however, we analyzed the first 5 CpG sites since they showed more than 90% matching identity with the promoter gene sequence (Rattus *HSD11B2* cDNA; GenBank acc. NM_017081) [30] which was analyzed with BiQ Analyzer software (Fig. 1).

### Real time PCR analysis

The levels of 11β-HSD2 and DNMT1 mRNA related to β-actin were determined by real time RT-PCR. Briefly, total RNA was extracted from 100 mg of placental tissue using TRIZOL reagent (Invitrogen, Life Technologies), and used to synthesize cDNA, using Moloney-Murine Leukemia Virus (M-MLV) reverse transcriptase (Promega, USA). The gene transcript levels were quantified separately using the LightCycler FastStart DNA Master SYBR Green I kit (Roche) following specific programs for each gene: 11β-HSD2, activation step at 95°C for 10 min followed by an amplification step with 40 cycles of 94°C 5 s, 60°C 6 s and 72°C 10 s; β-Actin, activation step at 95°C for 10 min followed by an amplification step with 40 cycles of 94°C 5 s, 55°C 6 s and 72°C 10 s; and DNMT1, activation step at 95°C for 10 min followed by an amplification step with 40 cycles of 94°C 5 s, 60°C 6 s and 72°C 10 s. The cDNAs were used as template for the real time PCR reaction, using specific primers for the respective gene Table 1. The results were calculated by the 2^−ΔΔC^T method [31].

**Table 1:** Sequence of primers for real time PCR.

Gen	Sequence	Melting Temperature	Nucleotide location
11β-HSD2 fw	5`-CCG GTT GTG ACA CTG GTT TTG-3`	60°C	389–409
11β-HSD2 rv	5`-GGG GTA TGG CAT GTC TCC TG-3′	60°C	788–807
β-Actin fw	5`-CCG TAA AGA CCT CTA CTG CA-3`	55°C	948–967
β-Actin rv	5`-AAG AAA GGG TGT AAA ACG CA-3`	55°C	1227–1246
DNMT 1 fw	5`-CGT GAC CTG CCC AAC ATA CA-3`	60°C	4367–4386
DNMT 1 rv	5`-GAA GAA GCC ATC CCA CTC CA-3`	60°C	4605–4624

Rattus *HSD11B2* cDNA (GenBank acc. NM_017081), Rattus norvegicus DNA methyltransferase (cytosine-5) 1 (Dnmt1) cDNA (GenBank acc. no NM_053354).

### Western Blotting

Concentration of 11β-HDS2 was determined by Western Blot using a primary anti-rat 11β-hydroxysteroid dehydrogenase type II polyclonal antibody (Millipore, CA) diluted 1:1000. For β-actin, we used a mouse primary antibody anti rat (Santa Cruz, CA, USA) diluted 1:2000. An anti-sheep IgG—peroxidase secondary antibody in a 1:10.000 dilution was used as secondary antibody (Sigma-Aldrich, St. Louis, MO, USA).

### Concentration of folates in placental tissue

Folate concentrations were determined in placental tissue (100 mg) using the ID-Vit Folic Acid kit (Immunodiagnostic AG, Germany) according to manufacturer instructions. This kit is based in a microbiological assay; the plates are covered with *Lactobacillus rhamnosus* that metabolizes FA. The presence of FA induces bacterial growth, which is measured at 610–630 nm [32].

### Statistical Analysis

Statistical analysis was performed utilizing the STATA 10 Program for MAC. Sample size calculation considering a power of 0.8 was based in our previous results [33]. Shapiro-Wilk test was used to assess the normal distribution of the dependent variables; only birth weight and placental weight were normally distributed. For these variables Student’s *t* test was used. To compare other variables among groups, the non-parametric Mann Whitney U test was used. For methylation studies Chi Square test and Bonferroni post-hoc test was used. Results are expressed as mean ± standard deviation (SD); statistical significance was set as *p* ≤ 0.05. GraphPad Prism software for MAC was used for creating figures.

## Results

Body weights of pregnant animals at day 0 and 20 (caesarean), litter, food and water consumption were similar in both groups. Birth weight of female fetuses from FA-supplemented mothers was 6% higher compared to females from control mothers (p = 0.003), with placentas weighing 8% less (p = 0.0004). However, no differences in male fetuses birth weight and weights of their placentas were found (p = 0.19) Table 2.

**Table 2:** Maternal and fetal characteristics.

	1—FA		8—FA	
	Mean	SD	Mean	SD
Female body weight at initiation (g)^a^	234	27	231	13
Female body weight at caesarean (day 20 of gestation) (g)	378	26	357	21
Birth weight (g)	4.65	0.4	4.83*	0.3
Male´s birth weight (g)^b^	4.84	0.3	4.94	0.3
Female´s birth weight (g)^c^	4.43	0.3	4.69*	0.4
Litter	13	2	14	2
Number of females/litter	6	2	7	2
Number of males/litter	7	1	7	3
Placental weight (g)^d^	0.51	0.04	0.47*	0.06
Placenta’s weight attached to females (g)	0.50	0.04	0.46*	0.06
Placenta’s weight attached to males (g)	0.51	0.05	0.49	0.06

Results are expressed as mean ± SD

^a^: n = 6 pregnant rats per group

^b^: n = 38

^c^: n = 32

^d^: n = 65

* *P*<0.05, significantly different compared to controls, Student’s *t* test.

**Fig 2:**
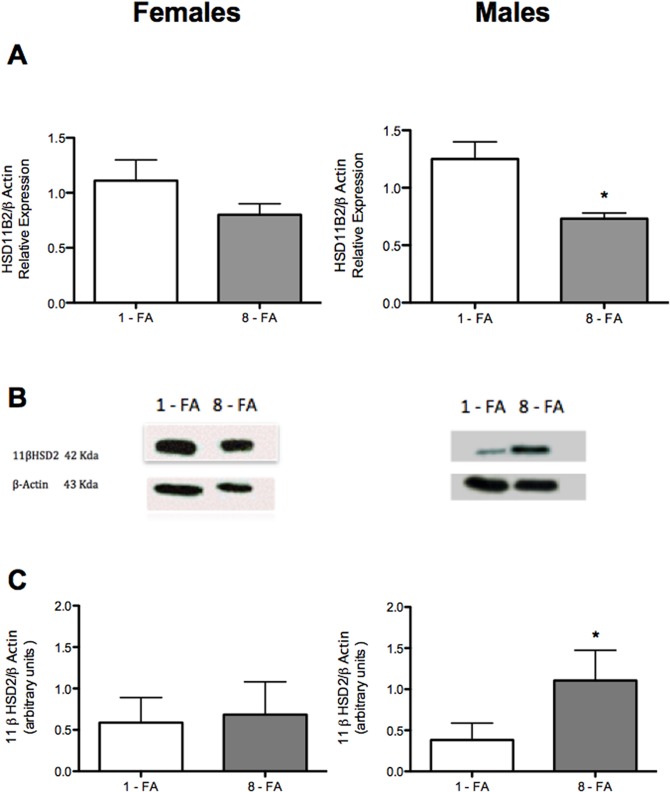
Expression of 11β-HSD2 in placentas attached to females and males. *HSD11B2* mRNA was determined by real time PCR as described in Materials and Methods. Results represent the HSD11B2/β actin ratio of duplicate experimental determination of 6 different biological samples of placentas attached to females and males from different litters (A). Western blot analysis for 11β-HSD2 protein expression in placentas attached to males and females (B). 11β-HSD2 western blot signals were determined by densitometry and expressed as arbitrary units related to β-actin (C). *p<0.05 compared to control; Mann-Whitney test.

Prenatal FA supplementation induced a decrease in mRNA of 11β-HSD2 in placentas attached to males compared to controls (*p* = 0.03), with no changes in the mRNA of placentas attached to females (Fig. 2A). In relation to protein levels, 11β-HSD2 concentrations in placentas attached to males were lower compared to females. Also, 11β-HSD2 concentrations in placentas attached to males of the FA- over supplemented group were higher compared to the control group (p = 0.006, Fig. 2B and C).

**Fig 3:**
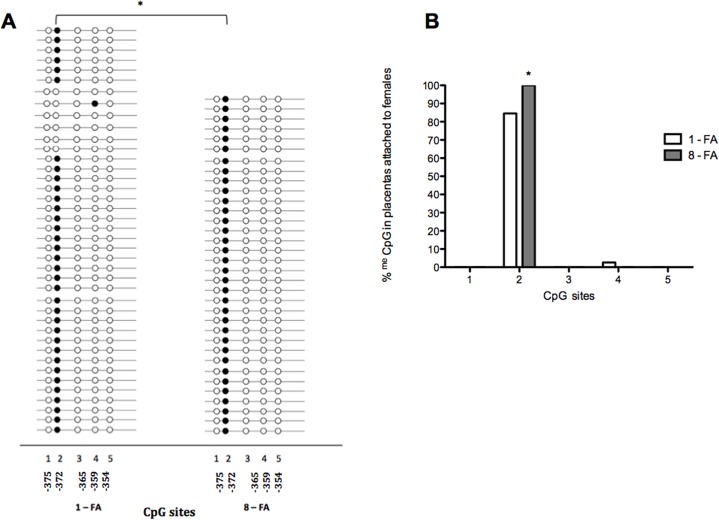
Methylation of *HSD11B2* in placentas attached to females in 5 CpG sites of the promoter region (−378 to −275). (A). DNA of six placentas attached to females per group was extracted (each placenta came from different litter). Results are expressed as percentage of methylation (%) (B). *p<0.05; Chi-square test.

**Fig 4:**
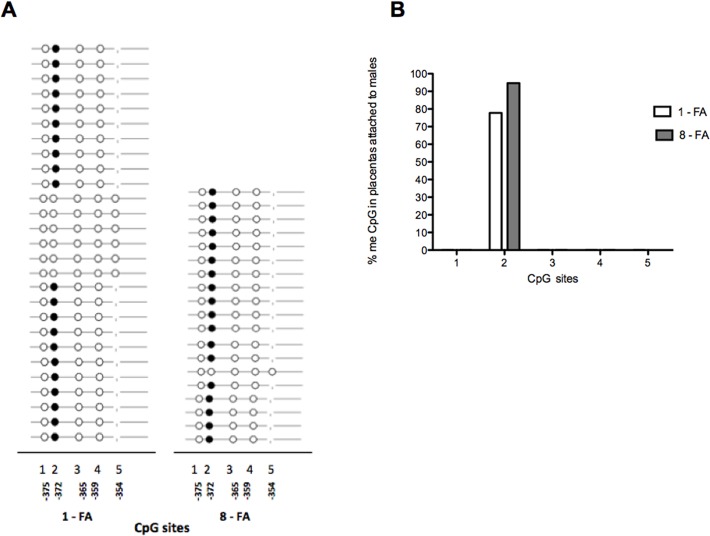
Methylation of *HSD11B2* in placentas attached to males in CpG sites of the promoter region (−378 to −275). (A). DNA of six placentas attached to males per group was extracted (each placenta came from different litter). Results are expressed as percentage of methylation (%) (B).

The five CpG sites of the *HSD11B2* promoter extracted from placentas were essentially not methylated in both groups, with exception of the CpG2, which was 80%, methylated, both in males and females. In FA-supplemented rats, the CpG2 methylation level increased slightly although only in placentas attached to females was the difference significant (113% increase) (*p* = 0.03) (Figs. 3 and 4).

The prenatal FA supplementation did not induce changes in the mRNA of DNMT1 both, in placentas attached to males and females Table 3.

**Table 3:** Placental DNMT1expression (mRNA) related to β—Actin.

	1—AF		8—AF	
	Mean	SD	Mean	SD
DNMT1 in placentas attached to males	1.02	0.2	0.73	0.2
DNMT1 in placentas attached to females	1.17	0.4	0.9	0.1

DNMT1 mRNA was determined by real time PCR as described in Materials and Methods. Results represent the DNMT1/β actin ratio of duplicate experimental determination of 6 different biological samples of placentas attached to females and males from different litters; p>0.05, Mann-Whitney test.

No differences in FA concentrations were found in placentas of the supplemented compared to control group Table 4, although a non-significant trend to higher folate levels in placentas attached to females was observed (*p* = 0.08).

**Table 4:** Concentration of folates in placental tissue.

	1—AF		8—AF	
	Mean	SD	Mean	SD
All placentas (nmol/g tissue)	1.55	0.3	1.75	0.6
Placenta’s attached to males (nmol/g tissue)	1.60	0.3	1.60	0.8
Placenta’s attached to females (nmol/g tissue)	1.50	0.3	1.90	0.4

Results are expressed as mean ± SD, (n = 6 placentas from different litters per group); P>0.05, Mann-Whitney test.

## Discussion

In this study, we found that FA supplementation of pregnant rats induces sex-specific changes that are related to placental GC metabolism. Prenatal FA oversupply induced a decrease in the expression of *HSD11B2* in placentas attached to males but not in those attached to females. Although a reduced placental expression of *HSD11B2* has been associated to a reduced birth weight [34], in our study, the weight of male fetuses was unaffected, most likely because the 11β-HSD2 protein levels in males were normal and even higher than controls (1-FA).

Conversely, only in female fetuses, the prenatal treatment with FA was related to a 6% higher birth weight compared to control group (1-FA). In this group, neither the 11β-HSD 2 mRNA nor the protein levels were affected by the FA-supplement. Previous studies have demonstrated that stress and maternal exposure to GC alters, in a sex-specific manner, the placental mRNA expression of several genes [35], and specifically the expression and activity of 11β-HSD2 [17–19]. In the present study, FA overexposure induced additional effects on 11β-HSD2 in placentas attached to males, probably at posttranscriptional level, since the concentration of 11β-HSD2 proteins increased despite that its mRNA decreased when compared with the control group (1-FA).

Kim et al. [36], studied the effect of FA fortification in female rats (8 mg/Kg) starting at 4 weeks before mating until day 20 of pregnancy. They found an increase in global DNA methylation level in placentas of hyperhomocysteinemic female rats and a correlation between global DNA methylation and FA levels in placentas of both sexes (*r* = 0.819). We did not evaluate global DNA methylation in the placental samples. Other differences include that females from that study were hyperhomocysteinemic and the FA supplementation began before mating.

It is unknown however, whether high levels of FA during pregnancy affect methylation of specific placental genes related to GC metabolism. Since gene expression may be associated to its promoter methylation, we studied whether the effect of prenatal FA supplementation was related to changes in the *HSD11B2* gene promoter methylation level. Analyzing the methylation status in five CpG sites—located at −378 to −275 bp from the starting codon of the *HSD11B2* gene promoter—we found that most CpG sites were not methylated except for site number two (CpG2), which was 80% methylated. At present, we do not know the relevance of a high methylation level in a single CpG site within its promoter sequence, nor whether it influences the transcription of that gene. After prenatal FA supplementation, *HSD11B2* methylation levels in specific CpG2 site increased slightly, but only in placentas attached to females the effect was significant. This suggests that the decreased expression of the *HSD11B2* gene found in placentas related to males is not related to the methylation level, at least in the CpG sites analyzed in the present study. Certainly, we cannot rule out that changes in the methylation status of other CpG sites located at the gene promoter may occur after FA supplementation.

On the other hand, in placentas attached to females of FA-supplemented pregnant rats, the methylation level of the CpG2 site increased by 13%. Such slight methylation change was not related to the *HSD11B2* expression, which did not change with FA supplementation in this group. At present we cannot evaluate the consequences of such differential effect of FA supplementation on the increased methylation level of one CpG site of the *HSD11B2* gene promoter.

It has been demonstrated that changes in the methylation of few CpG sites could determine changes in specific gene expression [22]. We could not demonstrate an association between increased methylation of CpG2 and *HSD11B2* expression. Previous studies suggest that slight changes in the methylation status of *HSD11B2* may alter the hypothalamus-pituitary-adrenal axis (HPA) [14,37,38]. A perturbation of the HPA axis, such as a prenatal chronic exposure to GC has been associated to IUGR, with long-term metabolic consequences involved in the development of chronic diseases. In the present study, we did not measured blood glucocorticoid levels, but GR expression in placentas of FA-supplemented animals did not change (not published results).

The association between placental methylation of the *HSD11B2* gene and intrauterine environmental factors contributing to birth outcome is unclear. Marsit et al. [22], in a study conducted in placentas of healthy newborn infants, not divided by sex, reported that the percentage of methylation in four CpG sites at the *HSD11B2* gene promoter region was associated with birth weight. More recently, Hogg et al. [39] did not find significant relationship between birth weight and *HSD11B2* promoter DNA methylation across normal birth weight ranges and in IUGR cases, suggesting that this issue is still under debate and results are probably highly dependent on the location of the CpG sites of the *HSD11B2* promoter in which the methylation analyses are conducted.

In a study with rats, IUGR has been related to epigenetic changes inducing a decreased *HSD11B2* gene expression and increased promoter methylation in the newborn kidney, which in turn leads to altered transcription factors binding [40]. In other study, by analyzing the *HSD11B2* gene expression in placentas from stressed pregnant rats, a higher methylation status in five of the 38 CpG sites analyzed as well as a lower expression of the gene was found. These findings were correlated with a high expression of DNMT1 and DNMT3a genes showing a direct effect of maternal stress during pregnancy on placental mRNA levels of the 11β-HSD2 enzyme through epigenetic mechanisms [15]. In our study, however, the increased methylation at the CpG2 site in the *HSD11B2* gene of placentas attached to FA-treated females was not associated with changes in the DNMT1 expression whose expression did not change by the FA over exposure (Table 3).

Previous studies in children have found a relationship between periconceptional FA use and methylation changes in *IGF2* gene [41], suggesting that FA supplementation during pregnancy may affect methylation of several specific genes that are related to fetal programming and development. In the present study, female offspring of FA supplemented female had 6% higher birth weight. Similar higher birth weights due to prenatal supplementation with FA has been previously demonstrated in rats [42,43]. In humans, higher birth weight has been reported in the newborns of mothers supplemented with 0.25 to 5 mg FA/day, compared to offspring of control mothers [44]; a 2% higher birth weight was found with two-fold increase in FA intake. Also, there is conclusive evidence showing a reduced risk of preterm and small for gestational age (SGA) delivery by FA and multiple vitamins consumption during pregnancy, indicating that FA may affect intrauterine growth [45,46].

Additionally, we found that placentas attached to females of FA- supplemented pregnant rats weighed less than placentas attached to prenatal FA-supplemented males (Table 2). This result could be an indication of enhanced placental function (defined as the fetal to placental ratio), as previously described by Wyrwoll et al. [47]. At present, we are not aware of other studies reporting changes in placental weights of mothers consuming FA supplements during gestation.

Kulkarni et al. [48] found that FA supplementation yielded an overall reduction of placental DNA methylation, only in the absence of vitamin B12, an essential vitamin implicated in the methyl cycle reactions. That study differs with the current study in that the diets given to pregnant rats contained normal concentrations of vitamin B12 and other contributors of methyl groups such as choline; the only variation in the diets of the present study was the oversupply of FA during the whole pregnancy period. Jiang et al., showed that choline intake in pregnant women can alter epigenetic state of cortisol-regulating genes and their expression in placenta [49]. All these results suggest that changes in DNA methylation associated to FA supply are evidenced only when the one-carbon metabolism is altered by an additional condition than the FA supply (e.g. hyperhomocysteinemia, deficit of vitamin B12, altered consumption of choline). For this reason, one of the main findings in this study was that the methylation of a single CpG site, located at the promoter of a specific gene, increased upon supplementation of FA in absence of additional nutritional alteration affecting the one-carbon cycle, and that this methylation change was sex-specific.

Kim et al [50], reported an increase in the placental concentrations of FA when female rats were supplemented with FA (8 mg /kg) 4 weeks before mating and thereafter, and paternal diets were also supplemented with FA (8 mg /kg) for 4 weeks. In the present study, however, we did not find increase in the FA concentration in placentas attached to either males or females. Although the experimental protocol of FA supplementation used by Kim et al., was completely different to that used in the present study, these results suggest the existence of regulatory mechanisms of the placental FA concentrations within which a reduction of FA to its active form (5-methyltetrahydrofolic acid: 5-MTHF) could be operating.

It is well known that environmental factors during pregnancy, like maternal diet and stress, can influence prenatal development and cause permanent structural and functional changes with consequences during adulthood [51]. In the present study, we have provided additional evidence showing that over supplementation with FA during gestation can affect placental 11 β-HSD2 gene expression in a sex-different manner, which in turn may result in unsuspected, sex-dependent physiological changes.

If these findings could be replicated in humans, it would be necessary to review the intake recommendations during pregnancy, not only for FA but also for other compounds acting as methyl group donors. Such compounds may be directly or indirectly involved in epigenetic modifications of specific genes, and may thus alter fetal growth and development with long-term metabolic consequences that ultimately result in undesirable chronic diseases.

## Conclusion

FA over supplementation during pregnancy in rats independently modifies both placental *HSD11B2* gene expression and methylation in one CpG site of the gene promoter (CpG2) in a sex-dependent manner.
